# Dataset on metabolomics profile of acute leukemia blood obtained by the NMR methods

**DOI:** 10.1016/j.dib.2017.02.035

**Published:** 2017-02-24

**Authors:** Alexander Bogaychuk, Andrey Zyubin, Anastasiya Lavrova, Galina Kupriyanova, Svetlana Babak, Mariya Dambieva, Maksim Demin, Anastasiya Borisova, Nikita Opryshko

**Affiliations:** a Immanuel Kant Baltic Federal University, A.Nevskogo St. 14, Kaliningrad 236016, Russia; b Research Institute of Phthisiopulmonology, Polytechnicheskaya Str. 32, Saint-Petersburg 194064, Russia

**Keywords:** Nuclear magnetic resonance, Acute leukemia, Metabolites, Spectral data

## Abstract

This article contains NMR (nuclear magnetic resonance) experimental data, obtained by the NMR Varian 400 MHz spectrometer (USA) which can be used for the metabolites identification in human blood. Data include analyzed NMR spectra of plasma proteins extracted from human blood of 24 patients (0–5 years old) with the confirmed acute leukemia diagnosis. Data can provide information about metabolites and their concentration in blood.

**Specifications Table**

**Table:** 

Subject area	*Physics*
More specific subject area	*NMR spectroscopy of blood proteins*
Type of data	*1 Table, 2 Figures*
How data was acquired	*NMR Varian 400 MHz spectrometer (USA)*
Data format	*Analyzed*
Experimental factors	Plasma proteins were extracted from patients blood using acetonitrile extraction and by solid phase extraction.
Experimental features	^1^Н NMR spectra were taken with Varian 400 MHz spectrometer (USA). Processing of spectra and identification of metabolites were performed using Chenomx software
Data source location	Kaliningrad, Russian Federation
Data accessibility	Data are presented with this article

**Value of the data**

– NMR analyzed spectra can be used for the metabolites identification.
– NMR analyzed spectra can be used for the calculation of metabolites concentration extracted by two different methods: acetonitrile method and solid-state phase method of samples preparation.
– Data obtained by the NMR can allow the choice of an appropriate method of sample preparation.

## 1 Data

**Fig. 1:**
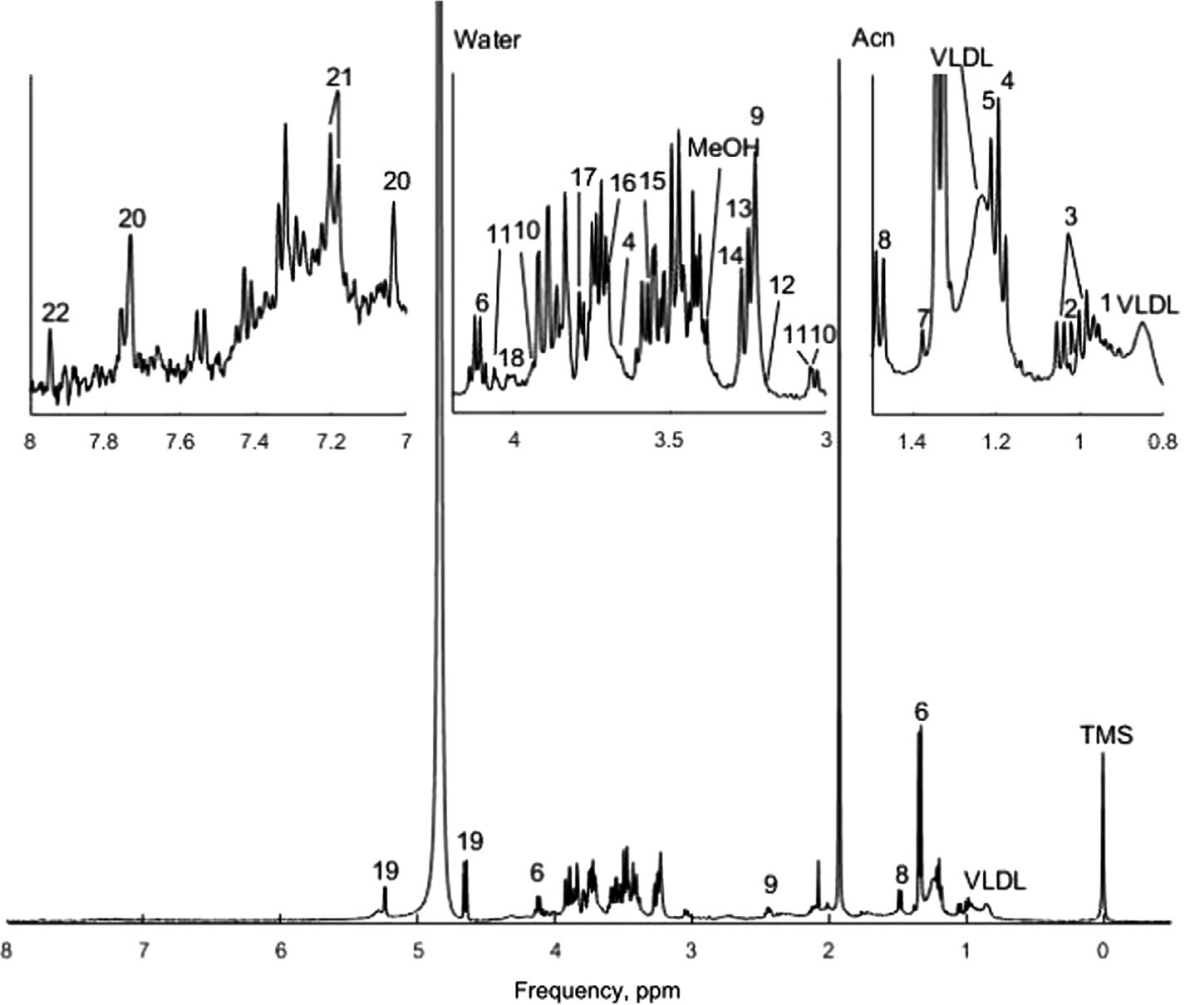
Data for metabolites, prepared with acetonitrile extraction. Each number on spectra peaks corresponds to the number of metabolite in Table 1.

**Fig. 2:**
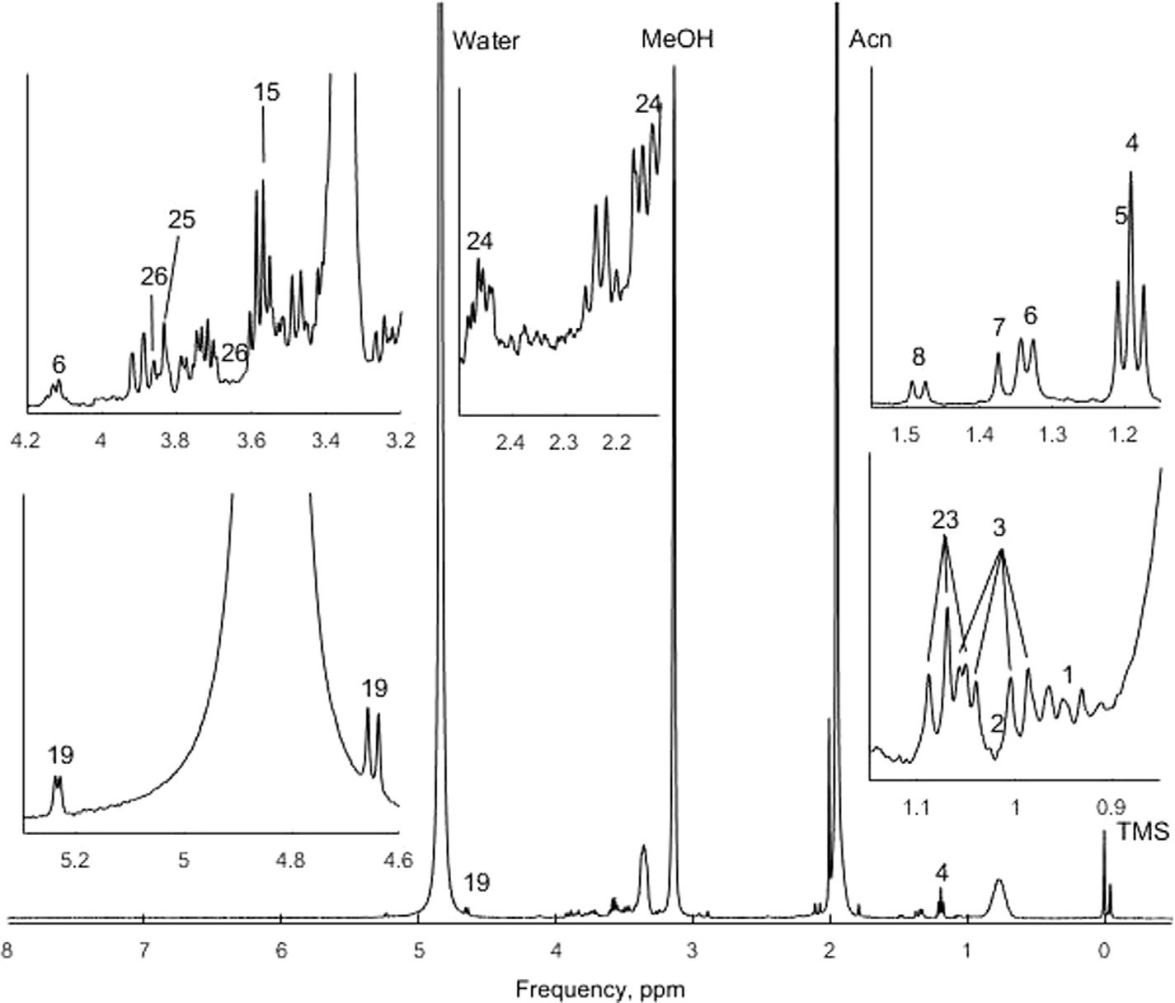
Data for metabolites prepared by the method of solid-state extraction. Each number on spectra peaks corresponds to the number of metabolite in Table 1.

The presented data include information on metabolites in blood and their concentration (Table 1), obtained by acetonitrile extraction (Fig. 1) and solid-state extraction (Fig. 2) methods.

## 2 Experimental design, materials and methods

The experiment׳s planning, design and data processing correspond to the protocols given in Refs. [1], [2], [3], [4].

### 2.1 Samples collection and storage

The data were taken from plasma proteins of 24 patients (0–5 years old) with the verified diagnosis of acute lymphoblastic leukemia and revealed severe side-effects after treatment. All patients were under pharmacotherapy and their adult representatives gave informed consent for the inclusion in the data processing and reporting. After one week of the treatment, venous blood was taken from all patients. The blood was transported to the laboratory on dry ice during one hour after blood sampling and stored at −80 °C. The blood was kept at room temperature before the analysis.

### 2.2 Sample preparation

#### 2.2.1 The method of solid-phase extraction

Reagents: methanol (Burdic & Jackson, Germany), 5% aqueous solution of methanol; acetonitrile (Sigma, Germany). Cartridges Agilent SampliQ Si-SAX 200, 60 Ǻ.

The plasma proteins were extracted using acetonitrile with following solid phase extraction. 500 µl of plasma 500 µl of acetonitrile was added and mixed on a vortex shaker for 3 min, then centrifuged at 10 000 rpm/min for 10 min. To the supernatant, in a volume of 500 µl, 600 µl of 5% aqueous methanol solution was added, then the sample in a volume of 1 ml was transferred to a pre-activated with 1 ml methanol sorption cartridge. Then the target components were eluted in 1.0 ml of 5% aqueous methanol.

#### 2.2.2 The method of acetonitrile extraction

Reagents: acetonitrile (Sigma, Germany).

500 µl of acetonitrile was added to 500 µl of obtained plasma, and then the solution was placed into the ice for 10 min, then it was mixed using a vortex-shaker during 30 s with the further centrifugation at 10,000 rpm/min during 10 min. Thereafter, 500 µl of a higher layer of the solution was taken out. After this, the rest of solution was centrifuged again at 10,000 rpm/min during 10 min to get the supernatant.

#### 2.2.3 Lyophilization

Previously, before drying, the samples have been frozen at −20 °C during 2 h. Lyophilization process was performed in LABCONCO Triad freeze-dried during 20 h at the vacuum pressure of 30 Pa. Sample temperature was maintained at −20 °C.

### 2.3 Description of the NMR experiment

^1^H NMR spectra were recorded via a Varian 400 MHz spectrometer (USA) with the precise frequency of the protons magnetic resonance equal to 399.85 MHz. Data were taken at the temperature of 298 K. To stabilize the resonance conditions, the dried samples were dissolved in 570 µl of 99,9% D_2_O. Also, 30 µl of tetramethylsilane (TMS) were added for the calibration of chemical shifts scaling and the determination of relative concentrations. The 60-degree RF pulse was used for the detection of ^1^H NMR. The signal was measured during 2.556 s. Spectral width was 6410.3 Hz, which is equivalent to 16 ppm (from −2 to 14 ppm). Recorded data sets contained 16384 counts. To increase the S/N ratio, a signal accumulation process was evaluated by using repetitive pulsing (256 pulses for each of all samples) with the relaxation delays equal to 15 s.

## Figures and Tables

**Table 1:** Identity, concentrations of metabolites.

Number of spectra	Metabolite	Concentration, mM/l	
		Obtained concentration	
		Acetonitrile extraction	Solid-state extraction
1	Leucine	5,40±0,40	1,40±0,40
2	Isoleucine	3,60±1,10	0,45±0,05
3	Valine	8,50±3,30	1,57±0,67
4	Ethanol	22,40±5,50	6,90±6,00
5	3-hydrobutirate	12,20±0,50	1,30±0,60
6	Lactate	66,40±34,60	6,20±3,10
7	2-hydroisobutirate	1,37±0,17	1,47±0,57
8	Alanine	13,33±6,93	2,47±1,37
9	Carnitine	4,53±3,33	–
10	Creatine	2,03±1,43	–
11	Creatinine	2,83±0,43	–
12	Choline	0,80±0,30	–
13	Betaine	2,30±0,40	–
14	Trimethylaminoxide	2,40±0,60	–
15	Glycine	15,13±3,23	11,73±2,83
16	Ethylene glycol	8,47±7,07	–
17	Guanidinoacetate	10,77±3,97	–
18	Fructose	8,75±1,25	–
19	Glucose	45,40±9,00	17,80±7,60
20	T-methylhistidine	2,23±0,33	–
21	Tyrosine	2,43±0,23	–
22	Xanthine	1,60±0,05	–
23	Propionate	–	1,37±0,87
24	Glutamine	–	4,47±2,27
25	N-nitrosodimetilamin	–	2,97±2,67
MeOH	Methanol	10,97±4,67	–
