# Supplementary material for: Dataset on metabolomics profile of acute leukemia blood obtained by the NMR methods
Source: Data Brief. 2017 Feb 24;11:479–83. doi: 10.1016/j.dib.2017.02.035 (PMC5338865; doi:10.1016/j.dib.2017.02.035)
Supplement: Supplementary file 1 — Supplementary material [file mmc1.pdf]

Dear Editors,

Authors declare no conflict of interests.

Sincerely yours,

Authors
